# Management of Splenic Abscess after Splenic Arterial Embolization in Severe Acute Pancreatitis: A 5-Year Single-Center Experience

**DOI:** 10.1155/2019/6069179

**Published:** 2019-07-01

**Authors:** Gang Li, Lin Gao, Jing Zhou, Bo Ye, Jingzhu Zhang, Cheng Qu, Lu Ke, Zhihui Tong, Weiqin Li

**Affiliations:** Surgery Intensive Care Unit (SICU), Department of General Surgery, Jinling Hospital, Medical School of Nanjing University, Nanjing 210002, China

## Abstract

**Objective:**

To describe the management and prognosis of splenic abscess after splenic arterial embolization in severe acute pancreatitis (SAP) patients.

**Methods:**

This is a retrospective observational study. From August 2012 to August 2017, SAP patients with infected pancreatic necrosis (IPN) who underwent splenic arterial embolization after massive hemorrhage of the splenic artery were screened and those who developed splenic abscess were included for analysis. The demographic characteristics, etiology, treatment of splenic abscess, and clinical outcomes of these cases were collected and analyzed.

**Results:**

A total of 18 patients with splenic abscess formed after splenic arterial embolization were included for data analysis. The median age of the 18 patients was 46 years. The etiologies included biliary AP, hypertriglyceridemic AP (HTG-AP), and other causes. Ten patients underwent minimally invasive percutaneous drainage only for splenic abscess while the other eight patients received splenectomy. One patient died due to uncontrolled infection and another patient died due to massive bleeding, and the remaining sixteen patients survived.

**Conclusion:**

The incidence of splenic abscess was high in patients requiring splenic arterial embolization due to massive bleeding. Our data showed that most splenic abscess could be successfully managed with minimally invasive interventions, and traditional splenectomy should serve as a backup treatment.

## 1. Introduction

Splenic arterial hemorrhage is one of the most serious and potentially lethal complications of severe acute pancreatitis (SAP), and the incidence of it is reported to be as high as 14.5% [1]. Angiographic and vascular embolization has been proven to be a safe and effective treatment option for these patients [2–4]. However, when splenic arterial hemorrhage is controlled, particularly with splenic arterial embolization, the probability of splenic infarction and/or splenic abscess formation increases significantly. Currently, surgical procedures remain the standard treatment for splenic abscess, and percutaneous drainage for splenic abscess is only reported in cases. In addition, studies regarding the diagnosis and treatment of splenic abscess caused by splenic arterial embolization in SAP patients are scarce in the literature.

In this study, we retrospectively collected a series of SAP cases undergoing splenic arterial embolization after bleeding to show the clinical characteristics of these patients and currently available treatment options.

## 2. Materials and Methods

### 2.1. Participants

In this study, all SAP patients complicated by infected pancreatic necrosis (IPN) admitted to Jinling Hospital, Medical School of Nanjing University, from August 2012 to August 2017 were screened for potential inclusion. Patients with splenic abscess who underwent splenic arterial embolization during the disease course were selected for analysis. The clinical data of all the study cases came from the prospectively collected AP database of our treatment center. The imaging data was collected from the Picture Archiving and Communication System (PACS) imaging system of Jinling Hospital. As a retrospective study, no ethics approval is required in our institute. To retrieve data from the electric database, we asked for an approval from the Acute Pancreatitis Database Management Committee. All the analyses were performed in compliance with the committee's regulation. Informed consent regarding data storage and publication was obtained from each patient who was recorded in the database during the hospitalization.

The diagnosis and disease severity classification of AP were defined according to the Revised Atlanta Classification [5]. The initial diagnosis of splenic abscess was based on the clinical symptoms and imaging findings. Abdominal enhanced computed tomography (CT) or ultrasonography examination was used in addition [6]. All CT images were reviewed by two radiologists, and the main manifestations of CT examination were triangular, conical, wedge-shaped, or irregular splenic low-density areas and no enhancement of the lesions. The main manifestations of ultrasonography examination were hypoechoic wedge-shaped regions in the spleen, and the blood flow signals in these regions could not be detected. The definitive diagnosis of splenic abscess required positive culture results of the drainage fluids or the histological evidence of surgically resected specimens.

### 2.2. Inclusion and Exclusion Criteria

The inclusion criteria for the study were (1) patients with a confirmed diagnosis of SAP based on the Revised Atlanta Criteria; (2) aged between 18 and 70 years; and (3) development of splenic abscess after splenic arterial embolization during the disease course. Patients were excluded if they met one or more of the following criteria: (1) they were in pregnancy, with malignancy or autoimmune diseases, etc.; (2) either splenic arterial embolization failed to control bleeding or IPN could not be managed by minimally invasive interventions and subsequently open surgery was conducted; and (3) no splenic abscess formation after splenic arterial embolization.

### 2.3. Splenic Abscess Management

The splenic abscess was managed with a two-step approach, which consisted of minimally invasive percutaneous catheter drainage (PCD) and splenectomy.

*Step one: PCD*. Image-guided PCD was well described in our previous study [7]. All PCD procedures were performed under the guidance of ultrasound or CT using the Seldinger technique. First, a percutaneous needle was inserted into the splenic abscess, avoiding the adjacent vital organs and viable splenic parenchyma. After that, the percutaneous sinus was dilated and a pigtail catheter (Bioteque Corporation, Taiwan, China) was inserted. After pus was acquired for culture, irrigation was started the next day with normal saline solution every 4  hour. If the clinical symptoms of the patient were gradually improved, the irrigation should be continued. If the patient failed to show clinical improvement in three days, he/she should be timely converted to splenectomy.

*Step two: Splenectomy*. Briefly, if a patient who underwent minimally invasive interventions showed persistent infective symptoms and/or deteriorated organ functions, he/she should be switched to splenectomy. A laparotomy through a left subcostal incision would be performed for these patients to completely remove the spleen and ligation of splenic blood vessels and postoperative indwelling abdominal drainage tube was inserted for irrigation.

### 2.4. Data Collection

The demographic data, diagnosis, and management as well as clinical outcomes of the study subjects were collected. The main clinical outcomes included the incidence of major complications (new-onset organ failure, sepsis, or severe local complications, including intra-abdominal rebleeding and subdiaphragmatic abscess) as well as in-hospital mortality, need for emergency surgery, ICU readmission after discharge, and duration of hospital and ICU stays.

### 2.5. Statistical Analysis

SPSS 22.0 statistical software package (IBM Analytics, Armonk, NY) was applied for statistical analyses. Data were presented as median with interquartile range (IQR) for continuous variables and absolute numbers and percentages for categorical variables. Student *t*-test or Mann–Whitney test was used for analyzing continuous variables, and the chi-square test was used for analyzing categorical variables. *P* < 0.05 was regarded as statistically significant.

## 3. Results

**Figure 1:**
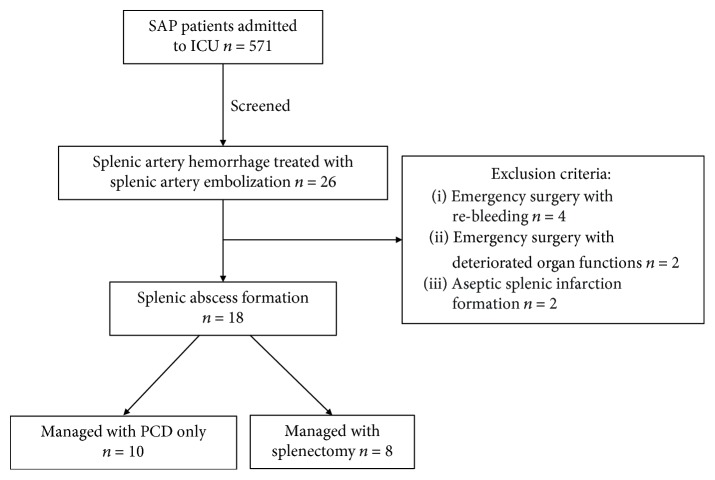
The flow chart of patients with severe acute pancreatitis.

There were 571 cases of SAP patients complicated by IPN admitted to Jinling Hospital screened from August 2012 to August 2017 in this study. Among them, 26 cases of splenic arterial hemorrhage were treated with splenic arterial embolization. Six cases of either rebleeding after embolization or aggravation of organ dysfunction necessitating emergency surgery were excluded. Besides, 2 cases of aseptic splenic infarction after embolization were excluded from this study (see Figure 1). Finally, there were 18 cases of splenic abscess formation after splenic arterial embolization included for analysis, among whom 10 cases were treated with percutaneous catheter drainage only and 8 cases required splenectomy. In the 18 patients, there were 14 males and 4 females, with a median age of 46 years.

The etiologies included ten cases of biliary pancreatitis, five cases of HTG-AP, and three other causes. The median SOFA score at admission was 7 (4.5, 8.5). The median time from disease onset to the diagnosis of IPN was 27 (15, 31) days and to the occurrence of splenic arterial bleeding was 53 (47, 74) days, and the median time from splenic arterial embolization to splenic abscess formation was 7 (4, 8) days. The main pathogens cultured in splenic abscess were *Escherichia coli* and *Klebsiella pneumoniae* (see Table 1).

After surgical or minimally invasive treatment for splenic abscess, 8 cases needed further intervention because of residual abscess or perisplenic residual infective lesions. Seven cases needed placement of thoracic drainage tube because of left intractable hydrothorax, of which reexamination of CT suggested the existence of subdiaphragmatic abscess. Four cases still suffered from bleeding after embolization, and among them, 3 cases were managed successfully by conservative treatment. Finally, one patient died due to uncontrolled infection and another patient died due to massive bleeding (see Table 2).

## 4. Discussion

Splenic abscess is a rare abdominal disease, and the literature has reported that the incidence of it in autopsied cases was 0.14~0.7% [8]. With the advancement in imaging technology and the growth in the number of patients with immune insufficiency, trauma, and tumor, the incidence of splenic abscess has gradually increased in recent years [9–11]. In addition, vascular disease, intravascular operation, surgical complications, and severe pancreatitis are also important causes of splenic abscess [2, 12–14]. Even so, intravascular embolization is still the first choice for pancreatitis-related splenic arterial disease [15]. In this study, we conducted a retrospective analysis of SAP patients complicated by IPN who underwent vascular embolization after splenic arterial hemorrhage. We found that only 2 out of the 20 patients with splenic arterial embolization did not develop splenic abscess, which indicated that the incidence of splenic abscess was high in this entity.

It has been reported that the main causes for splenic abscess are infective endocarditis, splenic trauma, abdominal infection, tumor, and immunosuppression [16–18]. In this study, we speculated that the direct causes for splenic abscess after splenic arterial embolization may include the immunosuppression state after long disease course (the median time from AP onset to embolization was 53 days), the presence of peripancreatic infections or abdominal infections, and the direct spread of pancreatic infection to the spleen.

The main clinical symptoms of splenic abscess are fever, pain in the left lumbar ribs, and the increase in white blood cells. However, these symptoms are not specific and there may be no overt symptoms in some patients with splenic abscess [19]. Consequently, splenic abscess is easily missed out or failed to be diagnosed in early phase. However, with the popularization of imaging examination, early diagnosis of splenic abscess has become easier to achieve [10]. In this study, as for patients with splenic arterial embolization who were expected to develop splenic complications later, we could diagnose splenic abscess in a relatively early stage with the assistance of timely imaging (B ultrasound and abdominal CT) results, and we found that the majority of splenic abscess could be diagnosed within one week.

The optimal management for splenic abscess is still controversial. It is traditionally thought that the effect of antibiotic therapy alone on splenic abscess was limited, and timely splenectomy was considered to be a reasonable choice for splenic abscess [9, 11]. Recently, with the improvement in minimally invasive interventions, B ultrasound or CT-guided percutaneous catheter drainage has gradually become an alternative to deal with splenic abscess. A retrospective analysis of 75 cases of splenic abscess found that percutaneous drainage was equally safe and effective compared with splenectomy, while splenectomy was still recommended for the splenic abscess with a larger diameter or multiple cavities [17]. Compared with the traditional splenectomy, minimally invasive intervention has significant advantages in preserving the function of the remaining spleen, reducing the traumatic stress response, and reducing the time of hospitalization [20]. For splenic abscess with multiple cavities, there were also reports showing that it could be successfully managed with percutaneous multiple tube drainage [21]. SAP patients are commonly accompanied by organ dysfunction, and the surgical treatments may cause further deterioration of organ functions or even lead to death. Our data showed that percutaneous drainage could be carried out under the guidance of bedside B ultrasound or CT to avoid the surgical stress. In view of the small number of cases in our study, more cases in multicenter studies may provide more convincing results for the optimal treatment of splenic arterial hemorrhage and splenic abscess after splenic embolization.

### 4.1. Limitations

Limitations of this study include the small number of the study cases and the retrospective nature of this study, which could easily cause selection bias.

## 5. Conclusion

The incidence of splenic abscess after arterial embolization was high in SAP patients complicated by IPN. For the management, PCD could serve as a primary choice of treatment with substantial successful rate and should be favored over upfront splenectomy with splenectomy only being considered if drainage fails or if the abscess has multiple cavities.

## Figures and Tables

**Table 1:** Baseline characteristics and clinical features of patients with splenic abscess after splenic arterial embolization.

Parameters	All patients (*n* = 18)
Median age (yr) (IQR)	46 (36-54)
Sex	
Male	14 (77.8%)
Female	4 (22.2%)
Etiology for pancreatitis	
Biliary	10 (55.6%)
Hyperlipidemic	5 (27.8%)
Others	3 (16.7%)
SOFA, median (IQR)	7 (4.5-8.5)
Median days from AP onset to IPN presentation (IQR)	27 (15-31)
Median days from AP onset to splenic artery bleeding (IQR)	53 (47-74)
Median days from splenic artery embolization to splenic abscess formation (IQR)	7 (4-8)
Treatment	
Percutaneous catheter drainage	10 (55.6%)
Splenectomy	8 (44.4%)
Pathogens cultured	
*Escherichia coli*	7 (38.9%)
*Klebsiella pneumoniae*	6 (33.3%)
*Enterococcus faecium*	1 (5.6%)
*Acinetobacter baumannii*	1 (5.6%)
Negative	3 (16.6%)

**Table 2:** Clinical outcomes of patients with splenic abscess after splenic arterial embolization.

Indicators	All patients
Need further intervention	8 (44.4%)
New-onset organ dysfunction	4 (22.2%)
Subdiaphragmatic abscess	7 (38.9%)
Rebleeding	4 (22.2%)
Mortality (%)	2 (11.1%)
Morbidity	
Median length of stay in days (IQR)	82 (44-118)
Median length of ICU stay in days (IQR)	69 (39-107)
ICU readmission after discharge	1 (5.6%)
Need for emergency surgery	1 (5.6%)

## Data Availability

The data in this study are available for other researchers to verify the results of our article, replicate the analysis, and conduct secondary analyses. Other researchers can send e-mails (e-mail address: njzyantol@hotmail.com) to contact us for obtaining our data.
